# Hospital Sunday: Who Were Its Founders and Originators?

**Published:** 1894-10-13

**Authors:** 


### HOSPITAL SUNDAY.

### WHO WERE ITS FOUNDERS AND ORIGINATORS?

### Mr. Henn's Own Case.

The Rev. John Henn writes from Heaton Chapel Rectory, Stockport:—

Someone has kindly sent me a copy of this month's The Hospital, for which I desire to express my thanks.

I have carefully read the article on pp. 451 and 452, and I must say that the loose manner in which the subject with respect to the originator of "Hospital Sunday" is treated has surprised me. I should have put down the paper in disgust had not the concluding sentence—"Historical accuracy is, however, essential in the interests of all, and that is why we have thought it necessary to place all the main facts on record in this article"—attracted my attention. The writer of the article referred to, I regret to say, has not been accurate in some of his quotations and references, and therefore his conclusions must necessarily be at fault. There is, *e.g.*, a quotation from the letter of an anonymous correspondent in the *Standard* on September 30th, 1880. That letter is not correctly quoted, and the last paragraph, which perhaps you will kindly allow me to transcribe, is omitted. It runs thus: "On the formation of a committee, with the Lord Mayor at the head, Canon Miller joined that committee, and, I believe, worked on it till his decease. But he was in no sense 'the founder of Hospital Sunday,' the idea of simultaneous collections for hospitals and dispensaries having been carried out in this city (Manchester) as far back as 1792, and revived eleven years ago by the present Rector of St. John's in the form which has so universally commended itself to popular approval."

The writer follows on by saying, "Despite the precise refutation set up in 1880 in regard to this claim in favour of the Rev. John Henn," &c. May I ask where the "precise refutation" is to be found, and by whom it was made?

Fault is then found with a letter written by (not "Mr. George Huntingdon," but) the Rev. George Huntington, Rector of Tenby, which appears in the *Standard* of June 10th, 1884, in which Mr. Huntington produces what he calls "indisputable evidence," &c., and the writer then proceeds to quote Mr. Huntington's words incorrectly, leaving out the most important part altogether. The writer's quotation from the inscription is this—"in recognition of his services for upwards of four years as secretary of the Manchester and Salford Hospital Sunday Movement." Mr. Huntington's words of the inscription, which is the correct version, are—"in recognition of the eminent services rendered by him as the originator and for upwards of four years as the honorary and acting secretary," &c. "Historical accuracy" requires that if a quotation be made it should be correctly given, together with the context, on which everything may depend.

The writer considers that he has given an answer to my question in my letter to *Forward* for last month, "Did Birmingham ever use the title of 'Hospital Sunday' before 1870?" By referring to a letter from the Rev. J. W. Marshall in the *Standard* of June 11th, 1884, in which (speaking of the Birmingham effort) he says, quoting from the Birmingham Red Book, "it appears that the first Hospital Sunday in that town occurred in 1859, just ten years before Mr. Henn's services were called into requisition." This, however, is no answer at all, because, writing as he did with an express object in view, and even apart from this consideration, he would naturally in such a place use the popular term for the collections, as it had then been used by others as well as ourselves continuously for fourteen years.

Further on there is a quotation from Chapter 10, Volume 3 of "Hospitals and Asylums of the World" (Scientific Press), in which it is stated that "Hospital Sunday" is a name always popularly used in Birmingham to describe these collections. Of course it is! These words were penned most probably three, or, at the most, six years ago; and it is twenty-five years since the popular name, which attracted such a degree of popular attention, became familiar. This also is no answer to my question—"Did Birmingham ever use the title of 'Hospital Sunday' before 1870?" Naturally, although Birmingham has not even yet discarded its original title of "Periodical Collections for Local Charities"—at least one of which is not a hospital as the word is usually understood—everybody since 1870 has used the shorter and more euphonious term of "Hospital Sunday." But I think I have some evidence that Hospital Sunday was a name new to Birmingham even subsequent to 1870, after we started "Hospital Sunday." In the Birmingham *Daily Post* of Monday, October 27th, 1873, there is an article which begins thus: "Yesterday, *what is now well known as Birmingham Hospital Sunday*," and so forth. (The italics are my own.)

Such an expression, I take it, refers to the new name which they had adopted, and not to the collections which had already been annually made for fourteen years. How, then, can the writer of your article be "historically accurate," when he says "the origin of 'Hospital Sunday' is therefore narrowed to Birmingham?"

I shall be glad if anyone can point me to any definite place in the newspapers, or elsewhere in print, where the name of "Hospital Sunday" was used before we used it in Manchester in 1870, when we started the movement for our hospitals and dispensaries.* Till that is done, Manchester must still continue to claim the origin of the title of "Hospital Sunday" (and the honour of instituting the Hospital Sunday movement), which is the name that has caught the sympathy of all people in pretty nearly all English-speaking places, and which has been imitated in "Diocesan Sunday" and other charitable enterprises.

### Mr. Henn's Claims Exploded.

To properly understand the points raised by Mr. Henn our readers should refer to page 451 of The Hospital for September 1st, 1894. Something which is said to have happened at Manchester more than one hundred years ago, and which has since remained buried in oblivion, can have no practical bearing on the Hospital Sunday movement established in Birmingham in 1859 by Mr. Wright and Canon Miller. The Rector of St. John's wrote to Mr. Burdett in 1875, and referred him to Canon Miller as the original source of his information, and so disposed of any claim to originality made on his behalf by the *Standard's* correspondents in September, 1880, and now on his own behalf in these columns.

The points Mr. Henn attempts to make may, however, be set out in order, and disposed of finally by documentary evidence. They are, briefly:

1. (*a*) Where is the precise refutation to be found; and (*b*) by whom was it made?

(*a*) *In the columns of the* Standard, *and in Volume III. of Hospitals and Asylums of the World.* (*b*) *By the Rev. J. W. Marshall and others, and by Mr. Burdett.*

2. That Mr. Huntingdon's letters and words were incorrectly quoted in The Hospital of September 1st, 1894, page 451.

*We have referred to the letter of the Rev. J. W. Marshall in the* Standard *of June* 11*th*, 1884, *from which Mr. Huntingdon's words were taken, and find our quotation to be precisely correct. This is, however, a small point, and the difference in the two wordings is not in any case material, as our readers will see from Mr. Henn's quotations.*

3 (*a*) Did Birmingham ever use the title "Hospital Sunday" before 1870? (*b*) "Until some one can point to a definite place in the newspapers where the name of Hospital Sunday was used before we used it in Manchester, Manchester must still continue to claim the origin of the title Hospital Sunday."

(*a*) *The present writer can state of his own knowledge that the title Hospital Sunday was in common use in Birmingham in* 1866, *and subsequently.* (*b*) *If Mr. Henn will refer to a leading article in the* Birmingham Daily Post *of October* 28*th*, 1865, *he will find it commenced, "Were it necessary to preach a lay sermon on Hospital Sunday." Capital letters are used, and no quotation marks. In the following year the* Daily Post *report of the periodical collections (October* 29*th*, 1866*) incidentally uses the expression, "Yesterday Hospital Sunday of* 1866," *&c. No doubt, an exhaustive examination of the files of the Birmingham papers would show that the collections there were often referred to as Hospital Sunday.*

Thus Mr. Henn's extraordinary and surprising claims to laurels which rightly belong to those now dead are easily disposed of. May we not hope that in the face of his letter of July 19th, 1875, and in view of the precise answers we have given to his questions, that Mr. Henn, who has really done excellent work as Honorary Secretary to the Manchester Fund, will now, for his own credit and reputation's sake, give the honour to whom it is due, namely, to the late Mr. Wright and Canon Miller?—Ed. The Hospital.

* In "Burdett's Hospital and Charities Annual, 1894," on pages cclxix. and cclxx., there is a list of 69 towns where there is a "Hospital Sunday." The first column of the tabular statement contains the dates of the foundation, where they have been ascertained, and only in three instances are these dates anterior to 1870—Nottingham and Wolverhampton, 1869; and Sheffield, 1868. But none of these three towns called the day they observed "Hospital Sunday" till Manchester used the title. If I am in error, no doubt some one will correct me. In a good many of the other towns, I know that they copied our "Hospital Sunday," from the correspondence I had with them, and it was probably the same with the rest. It occurs to me here to ask this question—If Birmingham were the example which all these towns copied, how is the gap between 1859 and 1868 to be accounted for?

